# The use of biological seed coatings based on bacteriophages and polymers against *Clavibacter michiganensis subsp. nebraskensis* in maize seeds

**DOI:** 10.1038/s41598-019-54068-3

**Published:** 2019-11-29

**Authors:** Chad Kimmelshue, A. Susana Goggi, Rebecca Cademartiri

**Affiliations:** 1 Department of Agronomy, Iowa State University, Ames, Iowa United States of America; 2 Seed Science Center, Iowa State University, Ames, Iowa United States of America; 3 Department of Chemical and Biological Engineering, Iowa State University, Ames, Iowa United States of America; 4 Department of Material Science and Engineering, Iowa State University, Ames, Iowa United States of America

**Keywords:** Plant sciences, Plant physiology

## Abstract

Biological control of bacteria with bacteriophages is a viable alternative to antibiotics. To be successful, biological control bacteriophages must be stable when exposed to the environment. Stabilization can be achieved through incorporation of bacteriophages into polymers and stabilizers that will be coated onto the seed. For this study, bacteriophages against *Clavibacter michiganensis* subsp*. nebraskensis (Cmn)*, the causal agent of Goss’s wilt, were incorporated into polyvinyl polymers with alcohol, ether and pyrrolidone functional groups and coated onto maize (*Zea mays* L.) seeds. The objectives of this study were to evaluate polymers and stabilizers that can protect *Clavibacter michiganensis* subsp*. nebraskensis* (CN8) bacteriophages against dehydration during storage. Bacteriophages stability when coated on seed depended on the glass transition temperature (Tg), functional groups of the polymer, and the presence of stabilizers such as sugars and proteins. Polyvinyl alcohol (PVOH) provided the greatest stability for CN8 bacteriophages on seed when coatings did not contain a stabilizer. A possible reason for the greater stability of this coating is having a glass transition temperature (Tg) very close to ambient temperature. PVOH combined with whey protein isolate (WPI) maintained CN8 bacteriophage activity in storage for four months at 26 °C and seven months at 10 °C. This coating also significantly reduced bacterial loads in seedlings grown from contaminated seeds, without affecting seed germination. Bacteriophage-polymer coatings which are stable during drying and storage, and are compatible with biological systems, not only provide an alternative to traditional antibiotics in agriculture, but also provide options for food, environmental and medical applications.

## Introduction

Biological control of bacteria is crucial in areas were antibiotic use is restricted, and where bacteria are resistant to antibiotics or copper compounds^1,2^. Biological control is achieved, for example, with beneficial bacteria^3–6^, biological derived molecules^7–9^, and bacteriophages^10–13^. Compared to antibiotics or copper compounds, biologicals are more sensitive to environmental stresses including temperature, humidity, and ultraviolet (UV) radiation^14–16^. For their protection, biocontrol agents are often incorporated into powders, gels, or film coatings containing natural and synthetic polymers, sugars, proteins, and controlled amounts of water^17–19^. Biological coatings also have found applications in medicine^20–22^, food^23^, and agriculture^24,25^.

In agriculture, antibacterial seed coatings can control bacterial plant diseases by protecting the seeds from contaminated plant residue, contaminated soil, and contamination within the seeds. While most coatings include chemicals like copper compounds and antibiotics, new biologically-based coatings are increasing in importance. Biological coatings can be more selective, which preserves the soil microbiome, and are compatible with organic practices. Successful biological seed coatings include natural oils, plant extracts, beneficial microbes, and bacteriophages^26,27^. For example, bacteriophages coated on seed or the soaking of seed in a bacteriophage culture reduced Stewart’s wilt in maize seeds^28^; reduced seedling rot and blight in rice seeds^29^; and improved nodulation and nitrogen uptake in soybean^30^. All of these bacteriophages were coated on seeds without polymers or stabilizers.

In general, there are three classes of bacteriophage coatings with varying levels of bacteriophage protection: unprotected bacteriophages^31,32^, protected bacteriophages applied as solutions^33^, and protected bacteriophages incorporated into gels or powders before coating^34^. The protected bacteriophages are generally more stable to environmental stresses. Research shows that bacteriophages applied to tomato leaves were more stable to drying and UV radiation when formulated into a coating with skim milk^35^, while bacteriophages incorporated into alginate gels were more stable at pH 2.5 than those in solution^36^. Adding sugars or skim milk to alginate gels before drying increased bacteriophage stability^37^, while whey protein isolate films maintained bacteriophage activity for five months^38^.

Coatings not only stabilize the biocontrol agent, but also the entity to be protected. For example, in agriculture polymers are used to protect seeds in many crops. These polymers provide a barrier between the seed and chemical treatments reducing phytotoxicity, and separate multiple treatments from each other, reducing negative interactions. Bacteriophages – polymer complexes can be water soluble which allows the release of bacteriophages as biocontrol agents upon rehydration^39^. Polymers also improve treatment adhesion and plantability while reducing dust off^27^. Dust off is crucial in the final stages of testing seed treatments to ensure the treatment stays on the seed during handling and planting. However, seed coatings can inhibit seedling emergence^40^ and even be phytotoxic^41–43^. Due to these possible negative effects, it is necessary to test seed germination and vigor for all treatments and coatings, including biological control.

**Figure 1:**
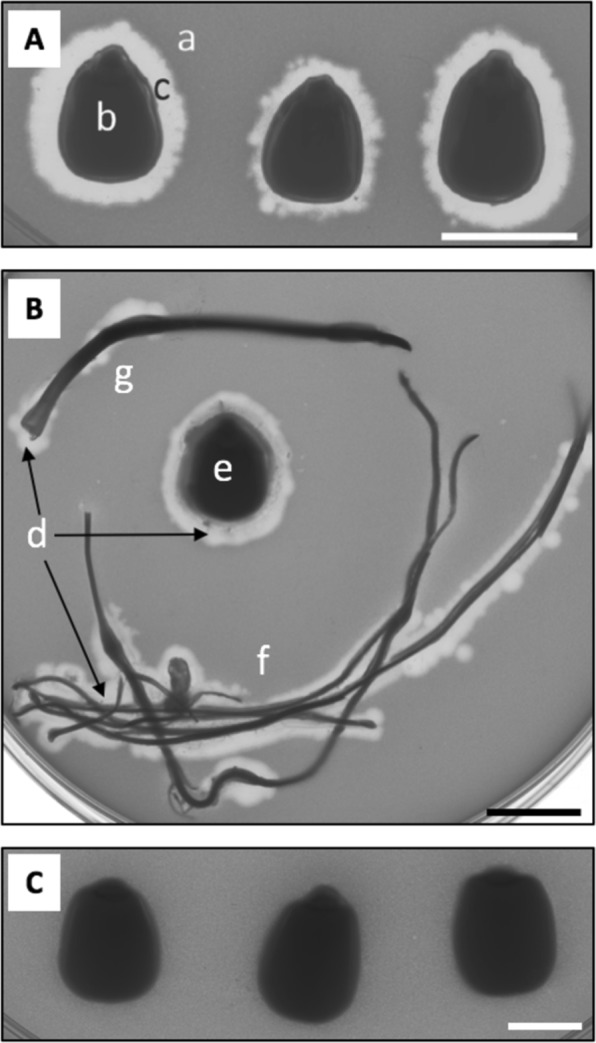
(**A**) Photograph of maize seeds (b) coated equally with CN8 bacteriophages (5.0 × 10^7^ PFU/mL) after incubation on a bacterial overlay of *Cmn* (a). Active CN8 bacteriophages led to lysis (c) around the seeds. (**B**) Visual confirmation (plaque formation) of CN8 bacteriophages presence (d) after germination around seed (e), roots (f), and shoot (g). (**C**) Control seeds coated with PVOH + WPI without CN8 bacteriophages showing no lysis around seeds. Lysis of *Cmn* only occurred when CN8 bacteriophages were added to the coating matrix. All scale bars: 10 mm.

In this study, we report on the ability of bacteriophages with polymers to act as biocontrol coatings on seeds. Specifically, we investigated the ability of CN8 bacteriophages to reduce *Clavibacter michiganensis subsp. nebraskensis* (*Cmn*) on maize seeds (Fig. 1). *Cmn* causes Goss’s wilt, a wide spread maize disease that may cause up to 50% yield loss^43,44^. More importantly, Goss’ wilt can be transmitted through infected seeds or plant residue in the soil^45^. Although the seed transmission rate is relatively low^46^, *Cmn* is an important pathogen to the seed industry because of phytosanitary restrictions to worldwide seed shipments, and to prevent the spread of *Cmn* into new areas. The objective of this study was to stabilize CN8 bacteriophages in different polymer coatings, to determine bacteriophage survival on the seed and their effect on seed germination, and to assess the feasibility of using this biocontrol seed coating to prevent the spread of *Cmn* through infected seed.

## Results

### Drying of CN8 bacteriophages into biocontrol coatings

**Figure 2:**
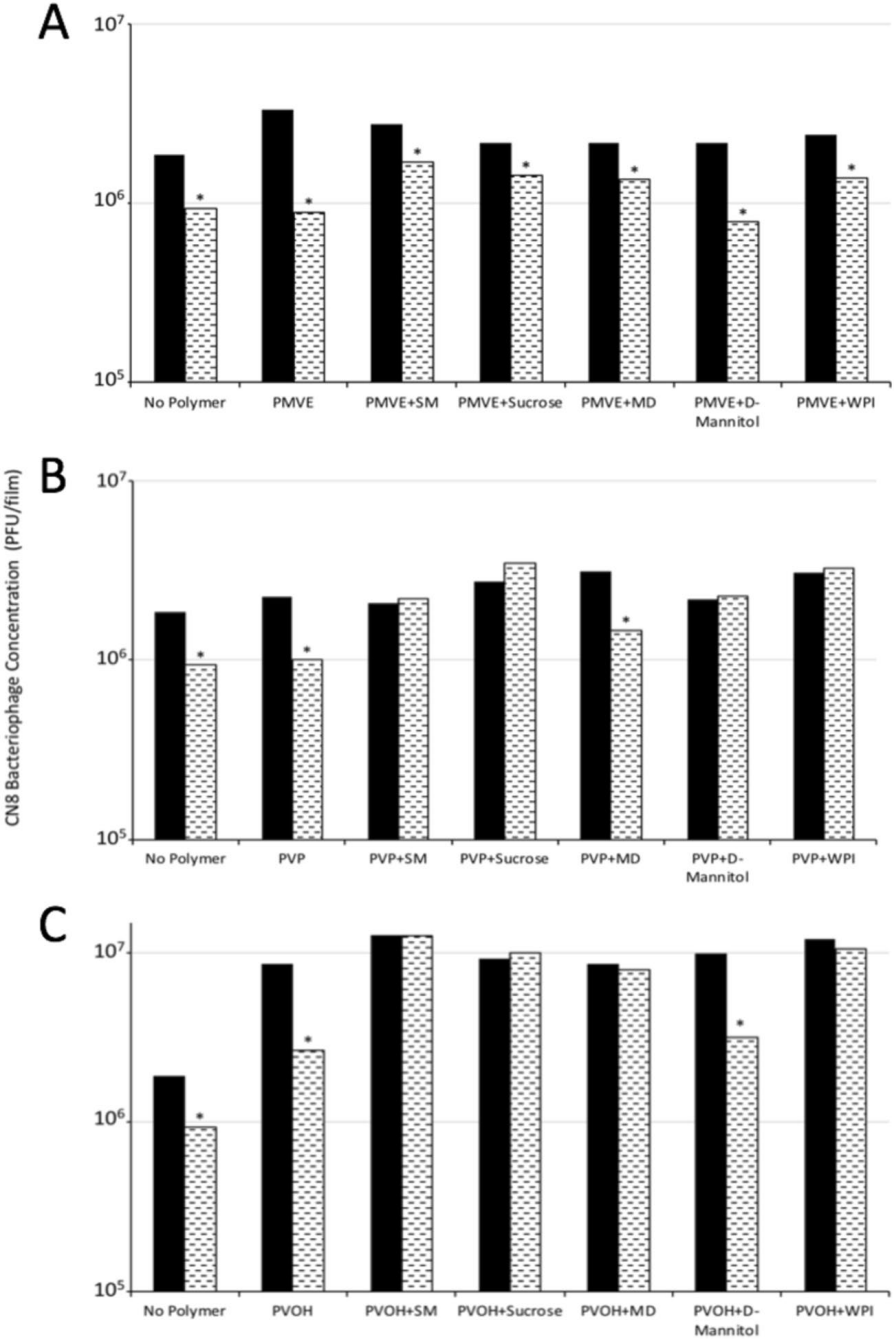
CN8 bacteriophages plaque forming units (PFU) in biocontrol films from different polymer (PMVE (**A**), PVP (**B**), and PVOH (**C**)) and stabilizer combinations before (black bars) and after drying (spotted bars) as polymer films on plastic. A mean difference in PFU concentration before and after drying is denoted by a star. Mean differences were determined via a t-test at a significance level of 0.05. Abbreviations: PMVE – Polymethyl vinyl ether, PVP – Polyvinylpyrrolidone, PVOH - Polyvinyl alcohol, SM – Skim Milk, MD – Maltodextrin, WPI – Whey Protein Isolate. Glass transition temperatures: PMVE (−82–22 °C)†, PVP (54–86 °C)†, PVOH 35–85 °C)^†^, SM (39–46 °C)§, Sucrose (28–70 °C)¶, MD (112–180 °C)‖, D-Mannitol (10–18 °C)‡, WPI (90–106 °C)§. Glass transition temperatures from †^89^‡^90^§^52^¶^53^‖^54^.

CN8 bacteriophages were stable after air-drying on plastic surfaces at ambient temperature (Fig. 2). Their stability depended on the polymer and stabilizer used. Eight of the eighteen coating combinations maintained the number of active bacteriophages during the drying process (indicated by treatments not containing a star above its bar in Fig. 2). Active CN8 bacteriophages were dried in the presences of PMVE, PVP and PVOH, and concentrations decreased between 0.3 and 0.5 log compared to the original liquid sample. Adding stabilizers improved the retention of active bacteriophages in the dry films. In the more flexible PMVE films, most stabilizers led to a 0.2 log decrease (D-mannitol log 0.4) in bacteriophage activity. In PVOH and PVP films, those including stabilizers led to the best protection and active bacteriophage concentrations in these films remained the same as those in solution (before drying). Only film combinations of D-mannitol/PVOH and maltodextrin/PVP did not maintain bacteriophage activity compared to the original liquid sample (Fig. 2).

**Figure 3:**
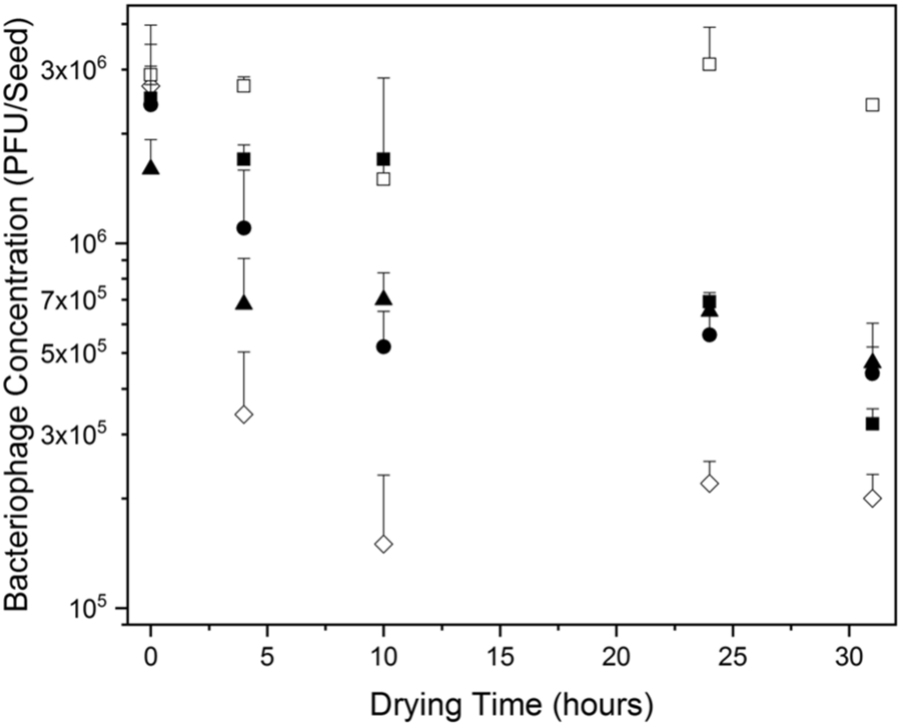
Concentration of active bacteriophages coated on maize seeds decreased with drying time depending on coating formulation. Open square: PVOH and WPI, closed square: PVOH, closed circle: PMVE, closed triangle: PVP, open diamond: bacteriophages in buffer. Error bars: two standard deviations. Only positive error bars shown for clarity.

Similar results were observed when coating bacteriophages on seeds. All CN8 bacteriophages dried on seeds showed infectivity (Figs. 1 and 3). Seeds placed directly on an overlay showed visual lysis of *Cmn* bacteria, which decreased after drying and storage (Fig. 1). While the size of the lysis area is related to the number of active bacteriophages for two dimensional materials, e.g., paper^47^, for three dimensional seeds inconsistent contact between the seeds and the overlay did not allow for quantification. Visual lysis was used only to confirm that bacteriophages in the biocontrol coatings can infect bacteria in a moist environment, similar to seeds planted in soil.

The number of active bacteriophages on the seed depended on the time of drying and the polymer and stabilizers used in the biocontrol coatings (Fig. 3). The number of active bacteriophages decreased rapidly during the first 10 h of drying (0.2 to 1.3 log) and then slowed to between 0 to 0.4 log. For most coating formulations, the number of active bacteriophages reached a plateau after 24 h. At 24 h, the number of bacteriophages applied in buffer had the largest decrease (1.1 log), followed by those in polymers (0.4 to 0.7 log) and those in PVOH/WPI, which showed no decrease.

When applied in commercially available seed coating formulations from BASF (Secompla 67c, FloRite 1197, FloRite 1706, and FloRite 1127) CN8 bacteriophages showed similar results to polyvinyl polymers. FloRite 1127 provided the best protection maintaining 1.1 × 10^6^ PFU/seed, followed by PVOH/WPI (9.67 × 10^5^ PFU/seed), and FloRite 1706 (2.8 × 10^5^ PFU/seed) (data not shown).

### Storage stability of CN8 bacteriophages coatings on seeds

**Figure 4:**
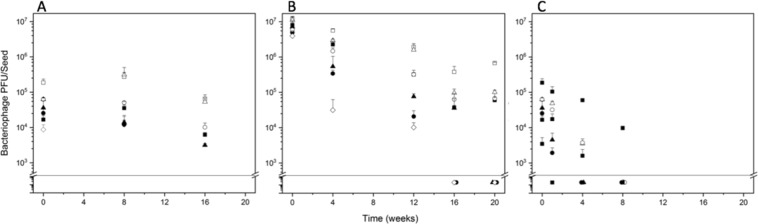
CN8 bacteriophages stability in biocontrol coatings on maize seeds stored at 4 °C (**A**), 10 °C (**B**) and 26 °C (**C**) for up to four months. The line indicates the limit of detection at approximately 600 PFU/seed. Values below that line are artificially spread to show all symbols. Open square: PVOH and WPI, closed square: PVOH, open circle: PMVE and WPI, closed circle: PMVE, open triangle: PVP and WPI, closed triangle: PVP, open diamond: bacteriophages only. Error bars: two standard deviations. (**A**,**B**) Only positive error bars shown for clarity.

With the multiplicity of infection (MOI) of 100 bacteriophages to a single bacteria cell^48^, we measured the storage stability of dry bacteriophages in polyvinyl-based biocontrol coating formulations on maize seeds stored at 4 °C, 10 °C, and 26 °C (Fig. 4) and at 26 °C for BASF coating formulations. The temperatures of 4 °C and 10 °C mimic ideal short-term storage conditions for seeds^49,50^, while 26 °C is commonly found in a farm warehouse^42^.

Bacteriophages infectivity after storage was enhanced when using lower storage temperatures. Coatings containing WPI maintained higher bacteriophages infectivity over storage time compared to polymer-only and buffer-only coatings, regardless of storage temperature (Fig. 4A). Bacteriophages infectivity in PMVE and buffer-only coatings decreased after week four, and was undetectable at week 16 of storage at 4 °C. A slow linear decline in bacteriophages infectivity was recorded for all other coatings after week four of storage at 4 °C, but some bacteriophages infectivity could still be detected at week 16. At 10 °C, a slow linear decrease in bacteriophages infectivity occurred for the first three to four months. The number of active bacteriophages at this temperature either decreased below the limit of detection (600 PFU/seed) or reached a plateau after seven months (Fig. 4B). Bacteriophages infectivity decreased below the detection limit in four months for PMVE, buffer-only coatings, while in PVP coatings infectivity lasted for five months. The loss of active bacteriophages at four months was more pronounced at 10 °C (1.5 to 2.1 log) than at 4 °C (0.9 to 1.0 log), with otherwise similar infectivity-reduction trends. CN8 bacteriophages infectivity further decreased when seeds were stored at 26 °C (Fig. 4C). After one month, only coatings with WPI or PVOH showed active bacteriophages present (0.5 to 1.3 log decrease). PVOH/WPI showed active bacteriophages at two months (1.3 log decrease), and decreased by 1.7 log when storage was extended to four months. With one hundred bacteriophages commonly used to kill a single bacteria^48^, this storage data indicates that the PVOH/WPI coating could eliminate up to 1.1 × 10^5^ *Cmn* bacteria when treatment first occurs and up to 3.8 × 10^3^ *Cmn* bacteria after storage (10 °C, four months) based on the number of bacteriophages that were initially coated on each seed.

Bacteriophage biocontrol coatings using BASF polymers provided different degrees of storage stability at 26 °C depending on polymer formulation. While Secompla 67c showed no active bacteriophages after five weeks, bacteriophage infectivity decreased approximately 1 log in FloRite 1197 and 1706, and only 0.5 log in FloRite 1127. At two months, the FloRite coatings continued to lose active bacteriophages at the same rate as seen in the five-week time point, with the exception of FloRite 1127, which only showed a 0.9 log decrease from the initial concentration. At three months, however, only FloRite 1127 showed any bacteriophages activity (2.0 log decrease), which was similar to that for PVOH/WPI at the same storage time and temperature.

### Biological control of *Cmn* on maize seeds

**Figure 5:**
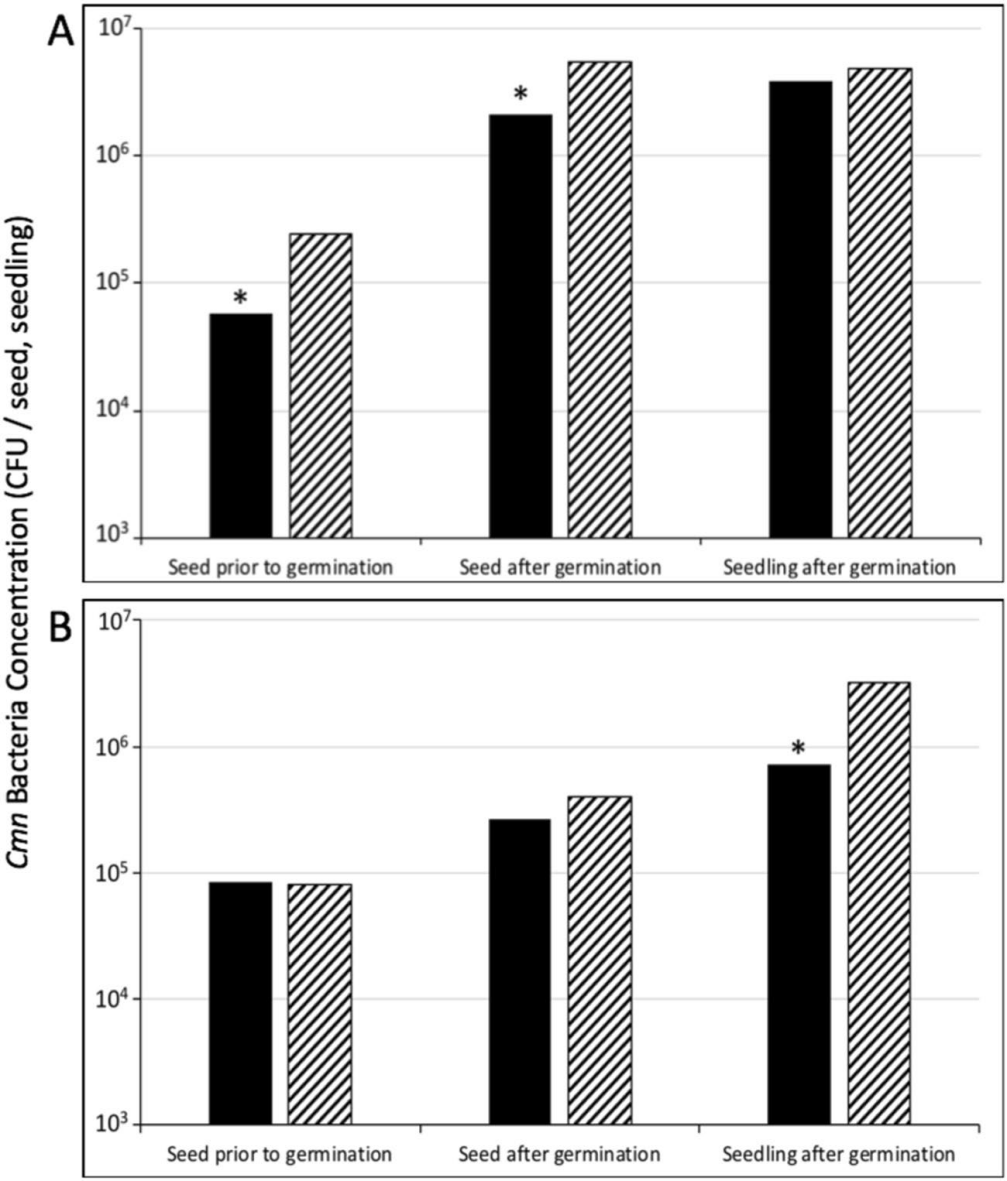
CN8 bacteriophages effect on *Cmn* mean colony forming units (CFU) in externally (**A**) and internally (**B**) *Cmn*-contaminated seeds before and after germination. Coated = black bars, non-coated = striped bars. Seeds were coated with CN8 bacteriophages in PVOH and WPI (5.0 × 10^5^ PFU/seed) and dried for 24 h prior to testing or germinating. Non-coated seeds were the untreated controls. Significance at the 0.05 level of probability is denoted by a star if the difference among the means was greater than the least significant difference (LSD).

Maize seeds were artificially contaminated, internally and externally, and coated with CN8 bacteriophages in PVOH/WPI. Bacteria and bacteriophage concentrations were determined before and after germination in sterile blotter boxes (Fig. 5). Visual confirmation of treated seeds showed that bacteriophages were present on the seeds and roots after germination for both internally and externally contaminated seeds (Fig. 1). Prior to germination, the bacteriophage coating significantly reduced the bacteria concentration on externally contaminated seeds by 1.85 × 10^5^ CFU/seed (76%). After germination, the CN8 bacteriophage coating reduced the *Cmn* concentration of externally contaminated seed by 3.17 × 10^6^ CFU/seed (58%) in the seeds and 9.67 × 10^5^ CFU/seedling (20%) in the roots and shoots (seedling), compared to the untreated control. Additionally, the bacteriophage coating reduced the *Cmn* concentration of internally contaminated seed after germination by 2.03 × 10^5^ CFU/seed (51%) in the seeds and 2.52 × 10^6^ CFU/seedling (78%) in the shoots and roots (seedling), compared to untreated control (Fig. 5).

### Germination and seedling vigor

Germination was not affected (*P* > 0.05) by biocontrol coatings or their components. Germination rates ranged from 97.5% to 99.5%. Seedling vigor, determined by the seedling dry weight test^51^, ranged from 42.1 mg to 49.2 mg per seedling. Analysis of variance indicated that seed vigor was not significant (*P* > 0.05); however, the overall variation among treatments suggested individual coatings produced more vigorous seedlings than the untreated control. A *t* test comparison between untreated seed and some of the seed coatings indicated significant differences. Seed coatings of WPI, and CN8 bacteriophages produced heavier seedlings (more vigorous) than the untreated seeds.

## Discussion

Bacteriophages can be protected during drying by reducing dehydration stresses^19,37,38^. These stresses can be reduced by controlling hydrogen bonding, glass formation, osmolarity, and the amount of residual water^52,53^. All polymers and stabilizers in this study allowed hydrogen bonding, had similar osmolarity (determined by the buffer), and were hygroscopic. Changes in CN8 bacteriophages infectivity in dry coatings were likely due to changes in their glass formation of the matrix as determined by its glass transition temperature (Tg) (Fig. 2). Poly(methyl vinyl ether) films with a Tg below ambient temperature were more flexible and provided less physical stabilization, than PVP and PVOH films with above ambient temperature Tg^54^. Bacteriophage stability in PMVE films improved when combined with above ambient temperature Tg stabilizers. The Tg probably was not the only mechanism responsible for CN8 bacteriophage stability. PVP and maltodextrin films, with the highest Tg^47^, did not improve bacteriophage stability (Fig. 2). Other potential factors described for bacteriophage stabilization include water exclusion from the protein surface^55^, and the stabilization of the folded state of proteins^56^. Proteins could potentially stabilize bacteriophages through hydrogen bonding with polar groups on the protein surface^57^, which all our polymers and stabilizers could do. On the seeds, the hydrophilic groups of the polymers could also interact with the carbohydrates of the outer layer of the maize seed^58^ adding another factor influencing CN8 bacteriophage stability after drying.

Not only the properties of the matrix can influence the number of active bacteriophages after drying, but also its thickness. CN8 bacteriophages belong to the order of Caudovirales and family of Siphoviridae^59,60^ with an icosahedral head (approximately 55 nm by 55 nm), a long non-contractile tail (approximately 220 nm) and tail fibers. Stabilization requires a dry coating that is at least as thick as the bacteriophage is long to allow full incorporation independent of orientation. Before drying coatings were approximately 63 μm thick, assuming equal distribution of active CN8 bacteriophages (10^6^ PFU/seed) and a surface area of 160 mm^2^ for the maize seeds^61^. After drying polymer and polymer/stabilizer, coatings were about 2 and 3 μm thick, respectively making them ten times thicker than the size of the CN8 bacteriophages. Buffer-based coatings would be significantly thinner, explaining their reduced stabilization of the bacteriophages.

After 24 h of drying, all polymer coatings showed similar retention of active bacteriophages, which was larger than the retention for bacteriophages coated only with buffer (Fig. 3). During long term storage, however, whey protein isolate (WPI) containing coatings provided significantly more protection and maintained more bacteriophages on the seeds than polymer-only coatings. These results were similar to those in WPI films^38^ and likely due to protein-protein interactions between the WPI in the film and the bacteriophage proteins^62,63^, and the protection of bacteriophage proteins and DNA from oxidative stresses^64^. Whey protein isolate provides an excellent barrier to oxygen and light, thus reducing these oxidative stresses^65^. WPI also protects the viral head of bacteriophages from desiccation, thus slowing bacteriophages of infectivity^35,66^. Our results were also similar to those seen for bacteriophages in electrospun PVP fibers^67,68^. In the fibers, there was no significant decrease in the bacteriophage’s infectivity directly after drying. During long-term storage, however, PVP fibers with sucrose or trehalose showed higher bacteriophages infectivity.

Cooler storage temperatures also improved bacteriophage stability and infectivity after storage. Temperature is the most important factor for bacteriophage stability^69^. Gonzalez-Menendez *et al*. reported a similar trend in which the storage time of alginate-encapsulated philPLA-RODI bacteriophages could be lengthened from one month to six months when storage temperature was reduced from 20 °C to 4 °C. When these same bacteriophages were spray dried in skim milk, their storage increased from six months at 20 °C to 12 months at 4 °C^70^. Storage temperature and relative humidity are as crucial for seeds^49,50^ as they are for bacteriophages. Corn seed is typically stored in a controlled environment at 10 °C and 50% relative humidity (RH). This temperature requirement is compatible with those of bacteriophages in our coating formulations and allows bacteriophage infectivity to remain active for at least 20 weeks storage. Our coatings could also protect bacteriophages coated on vegetable seeds because they are typically stored at 4 °C. The lower temperature would favor bacteriophages survival and infectivity after storage.

CN8 bacteriophages in PVOH/WPI coated on artificially contaminated seeds significantly reduced the number of bacteria present in the seedlings. Reduction depends on seed size and composition, pathogen location (internal or external), and transmission, amount of inoculum, and bacteriophage transmission to the site of infection^71–73^. In our study, pathogen location influenced the level of biocontrol. Our seeds coated with 5 × 10^5^ PFU/seed CN8 bacteriophages significantly reduced *Cmn* concentration on externally infected seed and seedlings from internally infected seeds. While our coatings did not completely eliminate *Cmn* from the seed or seedling, these seeds were intentionally artificially inoculated with a very high pathogen load to easily visualize bacteria mortality. Pathogen concentration in naturally infected seeds are much lower^43^. Our study indicates that bacteriophage-coatings have a similar biocontrol effect that a contact fungicide has on seed-transmitted fungi. To be effective, the bacteriophage-coatings must come in direct contact with *Cmn*. These coatings lowered the bacteria concentration on externally infected seeds, but not on internally infected seeds. The bacteriophages attached and lysed external *Cmn* decreasing its concentration, but did not penetrate into the seed. Similar results were reported in rice where bacteriophages decreased seedling rot incidence by eliminating B*urkholderia glumae and B. plantarii* bacteria from the surface of infested rice seed^29^. Even though our bacteriophage-coatings did not penetrate the seed, they effectively lysed *Cmn* in germinated seedlings. As seed germinated and *Cmn* was transferred from the seed to the seedling, bacteriophages released from the coatings interacted and lysed *Cmn*, reducing its concentration around the seedling roots and shoots.

While some biological control can negatively affect seed germination and seedling vigor^41^, other biocontrol coatings improved germination, e.g., of cucumber, areca palm, corn and canola seeds^74–76^. In our study, seed coatings had no effect on germination, presumably due to the high quality of seed used (all treatments >97% germination). However, seed vigor was positively affected by some of the biocontrol coatings. Whey protein isolate and CN8 bacteriophage seed coatings increased seed vigor over seeds without a coating. We speculate that this increase in vigor might be associated with a seed priming effect from the coatings. Seed priming is the controlled hydration of the seed to begin metabolic activities associated with germination, followed by dehydration^77^. Seed coatings used in these experiments are water-soluble and are applied onto the seed with small amounts of water. The water may rehydrate the seed, which is later dehydrated for storage. Other authors have reported improved seed vigor in seeds coated with polyethylene glycol, fish protein hydrolysates, *Trichoderma harzianum*, and water in seed priming^78–80^. These biocontrol coatings may not only provide disease management, but might also provide a positive effect on seed vigor.

## Conclusion

In conclusion, incorporating CN8 bacteriophages into polymer-based coatings in maize seeds significantly increased the stability of bacteriophages to dry storage without affecting the seed. While the focus of this study was on biological control of a plant pathogen with a seed coating, our unique findings on the influence of Tg, hydrophilic functional groups, stabilizers, and temperature on bacteriophages stability can be applicable to a wide range of biocontrol coatings on other surfaces. Future studies should investigate the use of bacteriophage coatings in the greenhouse, the compatibility of multiple bacteriophages in a single coating to control a wide range of diseases, and the use of bacteriophage coatings on other species. This understanding may allow us to prevent or treat a wide range of bacterial contaminations and diseases in agriculture.

## Materials and Methods

### Bacteria isolation and seed preparation

#### Bacteria and bacteriophages preparation and storage

*Clavibacter michiganensis subsp. nebraskensis (Cmn-91R)* was isolated from a maize field in Ames, Iowa, while CN8 bacteriophages were bought from the Félix d’Hérelle Reference Center for bacterial viruses of the Université Laval, Canada. CN8 bacteriophages were received as a glycerol stock containing 5.3 × 10^6^ PFU/mL. Bacteria were routinely cultured for 72 h in Nutrient Broth Yeast Extract (NBY) medium^81^ at 26 °C with shaking at 350 rpm (speed on incubating mini shaker to provide proper mixing) or on NBY agar^82^. In disease control experiments NBY base medium was supplemented with antifungal and antibiotic components (sCNS: potassium dichromate 0.02 g/L, thiabendazole 0.025 g/L, naladixic acid 4 mL/L, and cycloheximide 1 mL/L) to inhibit unwanted growth on the plate^43^.

CN8 bacteriophages were propagated on *Cmn* double agar overlays^83^. Briefly, 200 μl of CN8 bacteriophages in lambda buffer (5.8 g NaCl, 2.0 g MgSO_4_·7 H_2_O, 50 mL 1 M Tris-HCl pH 7.4, 0.1 g gelatin in 1 liter dH_2_O) were spread on NBY overlays (bottom layer: 1.5% agar, top layer: 0.5% agar) with 100 μl of *Cmn* (5.0 × 10^7^ colony forming units/mL) (CFU/mL) in the top layer and incubated for 72 h at 26 °C. After incubation, 5 mL of lambda buffer were added, the top layer and liquid removed, and centrifuged. The supernatant was filtered through a 0.2 μm cellulose membrane and the bacteriophage titer was determined using a spot assay with appropriate dilutions^83^. Bacteria and bacteriophages were stored at 4 °C.

#### Maize seed sterilization

A Goss’s wilt susceptible maize hybrid (Variety: H001872, Brand: 34C17, Lot: 1T34MA-3 MF) was purchased from Blue River Hybrids (Ames, Iowa). Maize seeds were surface sterilized, or surface and internally sterilized^84^ for all but germination and vigor testing. For surface sterilization, they were immersed in 50% ethanol for 5 min followed by 1% (w/w) NaOCl for 10 min. For surface and internal sterilization, seeds were immersed in 1% (w/w) NaOCl for 10 min, rinsed with sterile deionized water, and then soaked in sterile water at ambient temperature for 4–5 h followed by sterile water at 60 °C for 5 min. All sterilized seeds were rinsed with sterile water, transferred to sterile petri dishes and allowed to air dry overnight in a laminar flow hood.

#### Seed contamination

Surface and internally sterilized seeds were contaminated with *Cmn* (5.0 × 10^7^ CFU/mL) in NYB medium via vacuum-infiltration (internal contamination)^43^ or immersion (external contamination). For vacuum-infiltration, seeds were immersed in a 5-day-old culture of Cmn with one drop of Tween 20 under vacuum for 5 min. Contaminated seeds were surface sterilized in 1% (w/w) NaOCl for 10 min, rinsed with sterile water and left to dry in laminar flow hood overnight. For external contamination, seeds were soaked in a 5-day-old culture of Cmn at ambient temperature for 15 min on a shaker at 250 rpm. Seeds were air dried overnight prior to use.

The concentration of *Cmn* was determined by extracting the bacteria from the seeds. For internal contamination, 10 seeds were ground in a coffee grinder (KitchenAid Model: BCG111OB), followed by 2 min of vortexing in 10 mL of lambda buffer, and left to sit at ambient temperature for 1 h. For external contamination, the whole seeds were vortexed for 2 min in 10 mL of lambda buffer followed by 1 h rest. Concentrations were determined on sCNS agar.

#### Seed aging for germination inhibition

Seeds were aged using Accelerated Aging (AA) in accordance with the Assiociation of Official Seed Analysts (AOSA) Seed Vigor Testing Handbook^85^ to prevent germination of seeds on overlays. In short, a single layer of surface sterilized maize seeds was spread on an elevated screen, placed in an acrylic AA box containing 40 mL of water, and sealed with a tight lid. The boxes were placed into an AA chamber at 43 °C and 100% relative humidity for 96 h. Aged seeds were immersed in water at 65 °C for 10 min before sterilization and drying.

### Polymeric seed coating formulations

Coating formulations contained: Polyvinylpyrrolidone (PVP, 58,000 g/mol), polyvinylalcohol (PVOH, low molecular weight), or poly(methyl vinyl ether) (PMVE, 30% solution in water), or Secompla 67c, FloRite 1706, FloRite 1127, and FloRite 1197 from BASF (Ames, IA). Polymers were dissolved in deionized water (10% w/v) and stored at ambient temperature before use. Polyvinylalcohol required heating at 80 °C for 20 min to dissolve. Seed coating formulations contained 3.5% (w/v) or 3.5% (v/v) polymer, 5.0 × 10^7^ to 5.3 × 10^8^ plaque forming units/mL (PFU/mL) CN8 bacteriophages in water (94.5%) and 2% (w/v) stabilizers or 2% water, as appropriate. Stabilizers included whey protein isolate (WPI), skim milk, sucrose, maltodextrin, and D-mannitol. Coating solutions were prepared fresh before use.

### CN8 bacteriophages in polymeric seed coating formulation

The polymeric seed coating formulations described in the prior section (polymeric seed coating formulations) were tested for CN8 bacteriophage survival and activity in four separate experiments (dry polymer films, on seed storage stability, germination and vigor, and disease control). Each experiment was replicated three times, with the exception of germination, which was replicated two times before the next experiment was under taken.

#### Dry polymer films

CN8 bacteriophages in the seed coating formulations were dried into films and their activity was assessed by spot testing the dissolved films. In detail, 10 µl of seed coating formulation with 5.0 × 10^7^ to 5.3 × 10^8^ PFU/mL CN8 bacteriophages were added to 48 well plates and dried for 20–24 h in a laminar flow hood. Dry films were dissolved in 2 mL of lambda buffer and shaken at 300 rpm at ambient temperature for 4 h. CN8 bacteriophage titer was determined using a spot assay with appropriate dilutions.

#### Dry coatings on maize seeds

Sterilized maize seeds were coated with CN8 bacteriophages by immersion and dried in a laminar flow hood. In detail, 105 maize seeds were covered with 40 mL of coating formulation. After 15 min, seeds were removed, placed in sterile petri dishes, and dried for 20–24 h in a laminar flow hood. CN8 bacteriophage titer was determined using a spot assay with appropriate dilutions after dissolving the coatings. Three replications of ten seeds each were vortexed in 10 mL lambda buffer for 2 min, followed by standing at ambient temperature for 30 min. Vortexing CN8 bacteriophages (5.20 × 10^7^ PFU/mL) for 2 min in lambda buffer showed no change in concentration.

Coated seeds were placed embryo-side up on NBY overlays containing *Cmn* and incubated at 26 °C for 72 h, in order to test the infectivity of CN8 bacteriophages directly in the bioactive coating.

#### Storage stability of dry CN8 bacteriophage coatings on maize seeds

Coated seeds were stored at three temperatures and low humidity for up to 7 months. In detail, 100 coated and dried seeds were stored at 4 °C, 10 °C, and 26 °C with relative humidities of 23%, 23.2%, and 22.5%, respectively. Relative humidity levels were achieved with potassium acetate solutions of different concentrations^86^. The number of remaining active CN8 bacteriophages was determined every four weeks by dissolving the biocontrol coating in buffer and determining the concentration of bacteriophages in the solution via appropriate dilution and spot testing. While some bacteriophages remained on the seeds, a consistent number of bacteriophages was removed into solution.

### Seed germination and vigor

Germination tests were conducted using the rolled paper towel method^87^. In short, 50 seeds were placed down the middle of two moist paper towels (Anchor Paper Co., St. Paul, MN) and one moist paper towel was placed on top of the seeds. Paper towels were rolled up and held in place with a rubber band. Two replicates of 50 seeds were used for each coating, placed in their own bucket, and covered with a plastic bag secured with a rubber band. Buckets were randomly placed into modified food service carts (Lincoln Foodservice Products). Germination was performed at 25 °C in the dark, and seedlings were evaluated on day 7 using a standard scale^87^.

Seedling vigor was determined by separating germinated seeds from the roots and shoots, and drying the roots and shoots at 75 °C for 16–20 h^51^. Dry seedlings were weighed and the average weight per seedling calculated. Germination and vigor experiments were replicated twice by planting each replication 7 days apart.

### Seed Infection

Internally and externally contaminated seeds were treated with PVOH (3.5% (w/v)), WPI (2% (w/v)) and 94.5% CN8 bacteriophages (9.5 × 10^5^ PFU/mL) in water. Contaminated seeds were coated and dried following the same procedure as for the storage experiments. Coated and uncoated seeds were germinated at 25 °C for 7 days inside sterile boxes (6 × 9 inch hinged plastic box) containing two pieces of blotter paper (Anchor Paper Co., St. Paul, MN) moistened with 75 mL of sterile water. Each coating had 60 seeds separated equally into 3 boxes.

After 7 days, 10 seeds were separated from their roots and shoots. Separately, the seeds and roots/shoots were ground up and placed in 10 mL of lambda buffer or virucide solution (a ratio of 33: 70 mL was used. 33–7.5% black tea, and 70–10 mM FeSO_4_)^88^, vortexed for 1 min and left to sit for 1 h. One drop of chloroform was used to lyse bacteria before bacteriophage testing. Concentration of bacteria and bacteriophages were determined using spot tests on NYB sCNS agar and overlays, respectively. Roots, shoots and seeds were also directly placed on overlays to visualize plaque formation.

The seedling’s bacteria concentration was evaluated at 7 days to determine bacteriophage stability and survival, bacteria control, as well as its effect on germination.

### Statistical methods

Analysis of variance and T-tests for germination and vigor testing were conducted using SAS PROC GLM (SAS Institute Inc., 2012) to analyze seed treatment effects. Two-sample T-tests were conducted to compare treated versus untreated samples in disease control experiments, and polymer film drying using SAS PROC GLM. Equal variance was determined using Hartley’s F-max test, thus a pooled variance was used. Data was log transformed for disease control and polymer film t-tests and transformed back for presentation. Statistical analysis of storage stability studies was done using SAS PROC GLM.

## Data Availability

The datasets generated during and/or analyzed during the current study are available from the corresponding author on reasonable request.
